# Language, Reading, and Math Learning Profiles in an Epidemiological Sample of School Age Children

**DOI:** 10.1371/journal.pone.0077463

**Published:** 2013-10-14

**Authors:** Lisa M. D. Archibald, Janis Oram Cardy, Marc F. Joanisse, Daniel Ansari

**Affiliations:** 1 School of Communication Sciences and Disorders, the University of Western Ontario, London, Ontario, Canada; 2 Department of Psychology, the University of Western Ontario, London, Ontario, Canada; The University of Chicago, United States of America

## Abstract

Dyscalculia, dyslexia, and specific language impairment (SLI) are relatively specific developmental learning disabilities in math, reading, and oral language, respectively, that occur in the context of average intellectual capacity and adequate environmental opportunities. Past research has been dominated by studies focused on single impairments despite the widespread recognition that overlapping and comorbid deficits are common. The present study took an epidemiological approach to study the learning profiles of a large school age sample in language, reading, and math. Both general learning profiles reflecting good or poor performance across measures and specific learning profiles involving either weak language, weak reading, weak math, or weak math and reading were observed. These latter four profiles characterized 70% of children with some evidence of a learning disability. Low scores in phonological short-term memory characterized clusters with a language-based weakness whereas low or variable phonological awareness was associated with the reading (but not language-based) weaknesses. The low math only group did not show these phonological deficits. These findings may suggest different etiologies for language-based deficits in language, reading, and math, reading-related impairments in reading and math, and isolated math disabilities.

## Language, Reading, and Math Learning Profiles in a School Age Epidemiological Sample

Specific learning disabilities are a category of developmental disabilities characterized by difficulty learning in one or more areas despite otherwise typical neurological, physical, and emotional development, and adequate experiential and educational opportunities. Specific language impairment (SLI), dyslexia, and dyscalculia are childhood learning disabilities distinguished by the domain of the disability. SLI refers to a delay in the onset or development of oral language while dyslexia and dyscalculia refer to reading and math difficulties, respectively. 

Relevant findings reveal considerable heterogeneity within each disorder [1-5] as well as overlapping deficits between them [6]. However, the nature of the interrelationships between the various learning challenges faced by particular children remains poorly understood. One barrier to understanding children’s learning is that research has centered on children with impairments. A focus on academic learning patterns among children generally is important to understanding how common or unique certain learning profiles may be, which will ultimately lead to a better understanding of the cross domain learning challenges observed in children with impairments. A second impediment in this area is that research has been dominated by studies focusing on only single impairment profiles despite the widespread recognition of overlapping and comorbid deficits. As a result, it is difficult to determine whether the comorbidity represents a patterning together of different deficits, or is simply artifactual [7]. In order to better understand comorbidity, it is crucial that we adopt a more comprehensive approach to understanding children’s learning across a range of academic skills. To address this need, the present study aimed to explore learning profiles for language, reading, and math across a large sample of school aged children using an epidemiological approach.

### Comorbidities

Traditionally, SLI, dyslexia, and dyscalculia have been described as relatively specific deficits in respective areas. Children with SLI typically have impaired lexical skills (word knowledge) including late development of first words [8], and slow naming [9]. In addition, grammatical and syntactic knowledge has been found to be impaired in this group with particular difficulty in acquiring verb morphology [10,11]. Indeed, tense marking [12], nonword repetition [13], and sentence repetition [14] have been suggested as clinical markers of the disorder. Children with dyslexia often have difficulty with letter or symbol knowledge including slow and error-prone word reading [15]. Deficits in letter-sound knowledge impair both phonetic reading and spelling of single words. In addition, sentence level reading may be slow and laborious leading to difficulties understanding what has been read [16]. Difficulty with mapping phonology to orthography, tapped by phonological awareness and decoding tasks, are considered hallmarks of dyslexia [17,18]. Children with developmental dyscalculia have difficulty with basic calculations [19]. One of the hallmarks of dyscalculia is the persistent use of effortful calculation strategies (such as using finger counting) when typically developing peers have shifted towards retrieving the solutions to calculation problems from memory [20]. More recently, studies have shown that children with dyscalculia may also have difficulties with number sense, the ability to quickly understand, approximate, and manipulate numerical quantities [21,22]. It has been found that dyscalculia is associated with difficulty estimating the number of objects in a group [23], and comparing quantities [24,25], particularly when represented in a symbolic format (e.g. Arabic numerals). 

While pure forms of SLI, dyslexia, and dyscalculia occur, children identified with one of these learning disabilities often present with other co-morbid conditions. For example, SLI has been associated with high rates of speech production deficits [26], attentional difficulties [27], and motor discoordination [28]. Similarly, dyslexia often co-occurs with sensorimotor difficulties [29], attention deficits [30], and visual processing impairments [31]. Attentional difficulties have been reported also for dyscalculic groups [32,33], as have visuospatial processing deficits [34]. These significant and similar comorbidities have raised debate concerning whether these impairments stem from the same (e.g., [35]) or multiple deficits [36]. 

### SLI and Dyslexia

These three childhood learning difficulties also tend to co-occur with each other, although appreciably less is known about such comorbidities. The co-occurrence of SLI and dyslexia has received considerable attention. In a review of studies, McArthur, Hogben, Edwards, Heath, and Mengler [37] reported high incidences of language impairment among those identified as dyslexic (19% to 63%) and reading impairment among those identified with SLI (12.5% to 85%). As well, in two twin studies, rates of reading impairment were significantly higher than matched control groups in children with SLI [38,39] and family members of children with SLI [39]. Given that both oral language and reading skills rely on an intact language system, one view of the overlap between SLI and dyslexia is that they both stem from a language impairment affecting reading only in its mild form, and oral language and reading in more severe cases. Consistent with this view are findings of shared genetic effects across SLI and dyslexia [40-42]. However, recent evidence including separable genetic effects [41] and subtle dissociations in the oral language profiles of the two populations [43,44] suggest that this single deficit/severity view is too simplistic. 

Observations of the qualitative differences between SLI and dyslexic groups have led to the suggestion that both phonological and nonphonological dimensions of language must be considered in order to account for variations in language and reading development [45]. The prominent feature of the reading difficulty in classic dyslexia is a difficulty mapping between phonology and orthography stemming from a phonological processing deficit [46]. These children may struggle to decode unfamiliar words, but are able to comprehend those words once successful decoding has been achieved. Effortful decoding over many words, however, may lead to some breakdown in comprehension. While children with SLI often struggle with decoding, they also experience difficulty with reading comprehension even when they have accurately read a word [45,47]. It has been suggested that the oral language deficits in SLI impair comprehension of both spoken and written language [45]. Bishop and Snowling [45] have suggested a two-dimensional model to account for the unique and intersecting patterns of SLI and dyslexia including a common phonological deficit and an additional language-based (but nonphonological) impairment in SLI. 

### Dyslexia and Dyscalculia

Generally high rates of comorbidity have been reported for dyslexia and dyscalculia ranging from 17% [33] to approximately 60% [48,49]. As is the case for dyscalculia generally, research comparing dyscalculic groups with and without comorbid reading impairment is still in its early stages. Jordan and colleagues [50-52] found that children with comorbid dyscalculia and dyslexia performed more poorly than children with dyscalculia-only in exact calculation and solving story problems. No specific deficits were found for the dyscalculia-only group relative to the comorbid group. Consistent with these results are findings of similar patterns of impairment across a range of number processing tasks for dyscalculic groups with and without reading difficulties [24,53].

Nevertheless, some researchers have reported qualitative differences between groups with dyscalculia with or without dyslexia. Fuchs and Fuchs [54] replicated Jordan and Hanich’s [50] findings of poorer performance of dyscalculic groups with reading impairment than those without on untimed math facts and simple story problems. However, Fuchs and Fuchs did observe a disproportionate impairment for their dyscalculia-only group relative to the comorbid group on complex mathematical operations involved in story problems and real world problem solving. These results were interpreted as suggesting that the children with dyscalculia-only had more serious deficits in math procedures. Naming speed [55] and phonological and magnitude processing [56] have been examined in dyslexic-only, dyscalculic-only, and dyslexia/dyscalculia groups. These researchers found phonology-based impairments in the dyslexic groups irrespective of arithmetic status, and magnitude processing deficits in the dyscalculic groups irrespective of reading status. The deficits in the dyslexia/dyscalculia groups were considered additive in that they were equivalent to the impairments characterizing each respective specific group. The researchers hypothesized that dyslexia and dyscalculia have separable cognitive profiles that may co-occur. 

The high rate of comorbidity between dyslexia and dyscalculia may appear to undermine a strictly separable but co-occurring account of these disorders. An alternative view is that verbally based deficits may give rise to both reading and math impairments whereas a more domain-specific impairment related to number processing may underpin pure dyscalculia. Rourke and colleagues [57-59] have provided evidence consistent with this view. Children with comorbid dyslexia and dyscalculia had more difficulty with verbal than visuospatial neuropsychological tests whereas children with dyscalculia-only were more likely to struggle with visuospatial than verbal materials. Also in broad agreement are suggestions of subtypes of children with dyscalculia [20,60] including those with impairments in primary verbal working memory and conceptual knowledge, retrieval of facts from long-term memory, or visuospatial abilities. It should be noted, however, that there is a lack of evidence replicating the group differences [61] or supporting Geary’s subtyping (but see 54). Part of the problem across these research studies may be that consistency is lacking both in the criteria employed for identifying dyscalculia and dyslexia, and in the range of numerical, reading, and cognitive tasks investigated. 

### SLI and Dyscalculia

To our knowledge, no studies have investigated the comorbidity of dyscalculia and SLI. Nevertheless, a relationship may be expected. According to Dehaene et al.’s [62] Triple Code Model, three distinct systems may be recruited during mathematical processing including the quantity system (nonverbal), the visual system, and verbal representations (lexical, phonological, syntactic). Verbal representations are tapped, for example, during tasks involving fact retrieval or sentence-level processing. The reliance on verbal representations during some mathematical operations would lead to the prediction that children with SLI may experience difficulties in mathematical learning, and indeed, such a relationship has been reported. Children with SLI have difficulty acquiring early verbal numeracy skills such as logical operations and numerical representations but score at age appropriate levels on early nonverbal numeracy tasks such as numeral estimations [63-67]. Indeed, Kleemans et al. [67] found that phonological awareness, grammatical ability, and naming speed were significant predictors of early numeracy skills in children with SLI. It is clear that a systematic investigation of oral language, and verbal and nonverbal numeracy skills is needed to further our understanding of the relationships between developmental language and math impairments.

### The Present Study

The findings reviewed above provide strong evidence for the presence of important relationships between SLI, dyslexia, and dyscalculia. Nevertheless, work in this area has been hindered by inconsistencies in the criteria applied to define each learning disability, and in the tasks and analyses employed to investigate group differences. In addition, most studies have investigated the overlap between these disorders by focusing on small groups exhibiting only one or two of these disabilities. While informative, these studies cannot describe the overall pattern of variance and covariance in learning language, reading, and mathematics present in children generally, and in those struggling to learn specifically. It was the purpose of the present study to provide this crucial population perspective concerning SLI, dyslexia, and dyscalculia. In particular, this study is the first to consider learning patterns and challenges in language, reading, and math within subjects in a large developmental sample. The emerging patterns from this work can then form the basis for future research questions aimed at understanding corresponding deficits. 

A large, unselected group of school age children completed measures of language, reading, and math. One aim of the study was to examine the patterns of performance across measures using cluster analysis in which sets of observations are created using the dimensions of interest such that sets are more similar to each other within clusters than between clusters. We anticipated that general ability (i.e., performance across all tasks of interest) would yield several clusters reflecting ability levels (e.g., clusters identifying children who generally score in the low, average and high ranges across all tasks). Nevertheless, we were interested in differentiating patterns of performance, and so the analysis aimed to reveal additional clusters with unique profiles if they do in fact exist. Findings of clusters identifying solely language, reading or math weaknesses would be suggestive of specific and separable underlying mechanisms with comorbidity due to artifacts in the data. The presence of clusters with weaknesses in multiple areas would be reflective of potentially meaningful comorbidity perhaps suggesting a different pattern of core deficits. We also examined whether the same pattern of learning profiles would be found for those performing at the low end of the distribution. Findings of similar distributions would suggest that our cluster rates might be applicable to learning disabilities. 

A second goal of the study was to provide a preliminary validation of our clusters, and to explore cognitive performance differences across clusters using data available for a subset of the large, unselected sample. Findings that a particular cognitive profile characterizes both unitary and comorbid clusters would implicate similar underlying processes whereas different cognitive deficits for unitary and comorbid clusters might suggest separate underlying cognitive constraints. 

## Methods

The Nonmedical Research Ethics Board at The University of Western Ontario approved all procedures in this study.

### Participants

A total of 34 schools (including 5 rural schools) in the southwest region of Ontario, Canada were recruited to the study. All children in senior kindergarten through grade 4 in each of the schools were invited to participate in October of the school year, corresponding to an age range of 4 years;10 months to 10;10. Approximately 5967 consent forms were distributed of which 1605 were returned, signed by parents. Of these, 1387 children participated in the study (the remainder were either outside the age range, *n* = 61, or could not be screened within the time frame of the study, *n* = 157). Complete data sets were obtained for 1120 children aged 6;0 to 9;11 (Epidemiological Sample). Children under 6;0 were not included in the present study because they did not complete the reading screening measure (*n* = 178). Of those meeting the age criteria for the present study, 14 did not have complete data sets. By parent report, approximately 86% of the children were right handed, 85% spoke English as their first language, and 82% of mothers had at least some college or university education. It should be noted that these proportions did not differ from that of the full sample (*n* = 1605). Additional data were available for a subset of the Epidemiological Sample who participated in further studies with our research group (referred to as the Standardized Test Subsample, see below; *n* = 383).

### Procedure

All participants in the Epidemiological Sample completed a 10-minute screening protocol consisting of four tasks, *Sentence Recall, Math Fluency*, *Sight Word Reading Efficiency*, and *Phonemic Decoding Efficiency*. The Standardized Test Subsample completed a battery of standardized language, reading, math, phonological awareness, intelligence, and working memory tests (and other measures not reported here) in three visits occurring one week apart and within 6 months of the original screening. All tasks were administered individually in a quiet room in the child’s school by a trained research assistant.

### Screening Measures

All participants completed the screening measures described below.

#### Sentence Recall

The sentences were taken from Redmond [68] and consisted of 16 sentences each composed of 10 words (10 to 14 syllables) with an equal number of active and passive sentences. Although not standardized, this task has been found to have good sensitivity and specificity for identifying children with language impairment [69]. The sentences were presented in fixed order via a digital audio recording of an adult female speaker using headphones. Sentences were scored online by the research assistant with either a 2 (correct), 1 (three or fewer errors), or 0 (more than four errors or no response). 

#### Math Fluency

The *Math Fluency* subtest of the *Woodcock-Johnson III Test of Achievement* (WJ III; [70]) involves the rapid application of basic addition, subtraction, and multiplication procedures. Questions graded in difficulty were presented on an 11” by 17” sheet, and children were asked to complete the problems as quickly and accurately as possible for three minutes. The total number of problems completed correctly was counted. 

#### Reading Efficiency

The *Test of Word Reading Efficiency* [71] was administered. In the Sight Word Efficiency (SWE) subtest, children read as many printed words as possible in 45 seconds. In the Phonemic Decoding Efficiency (PDE) subtest, children read as many pronounceable printed nonwords as possible within 45 seconds. The total number of words/nonwords read correctly was counted for each subtest. 

### Standardized Test Battery

The Standardized Test Subsample was comprised of monolingual English speakers, and was selected based on criteria motivated by other studies focusing on children with impairments and on practical constraints. Briefly, standard score cutoffs equivalent to -1.3 *SD* (19.5 standard score points) below the full sample mean were set separately for each of the sentence recall (*M* = 103; *SD* = 18), phonemic decoding efficiency (*M* = 106, *SD* = 13), and math fluency tasks (*M* = 93, *SD* = 14), or for a pattern of low performance equivalent to -1.0 *SD* (15 standard score points) on more than one of these tasks. The cutoff point of -1.3 *SD* was based on previous findings of high agreement between clinician judgments of SLI and test scores of at least -1.25 *SD* below the standardized mean [72]. The cutoff for a pattern of low performance across tasks was narrowed to -1.0 *SD* due to practical limitations related to testing all the children meeting the corresponding criteria. For the applied criteria, standard scores were based on the present sample for the sentence recall, and on the published test norms for the remaining tests. No cutoff was set for the sight word efficiency test. 

The Standardized Test Subsample comprised all low performers, that is, all who scored below the cutoffs and who could be tested (186/255), and children who scored within the average range on all screening tasks and attended the same schools as the low performers (*n* = 193). Although the composition differs from that of the Epidemiological sample, the performance of the Standardized Test Subsample provides some indication of the characteristics of relevant learners when interpreted with caution. The information may be especially relevant to those with learning challenges because low and average scorers on the screening tasks were represented in roughly equivalent proportions in this Subsample. All participants in the Standardized Test Subsample completed the measures described below.

#### Language

Each child in the Standardized Test Subsample completed the four core subtests appropriate for the child’s age for the Composite Language Score (CLS) from the Clinical Evaluation of Language Fundamentals IV (CELF-IV; [73]). In the *Concepts and Following Directions* subtest, the child pointed to aspects of a picture following a spoken instruction. For *Recalling Sentences*, the child repeated sentences immediately after hearing them and for *Formulated Sentences*, created a sentence using a given word. Children under 9 years completed the *Word Structure* subtest involving completing a sentence with the grammatically correct word form, and those 9 years and over completed the *Word Classes 2* subtest involving identifying which two of four words have a related meaning.

#### Reading and Math

Two subtests from the WJ III [70] were administered to each child. In the *Reading Fluency* subtest, the child read a sentence and answered yes/no questions about the sentence. The child completed as many as possible in three minutes. In the *Calculations* subtest, the child was asked to complete mathematical operations. 

#### Phonological Awareness

In the *Elision* subtest of the Comprehensive Test of Phonological Processing (CTOPP; [74]), the child was asked to isolate and delete a phoneme from a word. For example, say ‘stop’, say it again without saying ‘t’.

#### Intelligence

The children completed the four subtests of the Wechsler Abbreviated Scale of Intelligence (WASI; [75]). The nonverbal intelligence subtests included *Block Design*, in which the child arranged blocks to match a model, and *Matrix Reasoning*, which involved choosing a picture to complete a pattern. The verbal intelligence subtests included *Vocabulary*, in which the child provided definitions, and *Similarities*, which involved identifying related pictures or describing similarities between words. 

#### Working Memory

Eight subtests from the Automated Working Memory Assessment (AWMA; [76]) were administered. Measures tapping phonological short-term memory involved immediate repetition of numbers or nonword forms (*Digit Recall, Nonword Recall*), and those tapping visuospatial short-term memory required recall of locations (*Dot Matrix, Block Design*). Verbal working memory measures involved recall of counts or final words after counting or processing a sentence, respectively (*Counting Recall, Listening Recall*), while those involving visuospatial working memory required the recall of location or orientation after identifying a different shape or mentally rotating an image, respectively (*Odd One Out, Spatial Recall*).

### Data Analysis

In order to compare performance across our screening tasks, we created our own normative scores based on our large, unselected Epidemiological Sample. To do this, raw scores from the screening measures were converted to z-scores within four age bands (6;0-6;11; 7;0-7;11; 8;0-8;11; 9;0-9;11) and then transformed to a standard score scale with a mean of 100 and a *SD* of 15. Standard scores for all of the tests completed by the Standardized Test Subsample were based on the respective published test norms.

In order to explore the patterns of unique learning profiles in our Epidemiological Sample, we completed a two-step cluster analysis appropriate for large samples (SPSS v. 17). The cluster analysis technique groups cases of observations into discrete subgroups based on their similarity across a set of chosen dimensions. The clustering procedure proceeds hierarchically so that smaller subgroups (clusters) are merged iteratively into increasingly larger clusters. We planned to explore the number of unique learning profiles by systematically increasing the number of clusters to be found by the solution until no further unique learning profiles were established. Additional tests available for the Standardized Test Subsample allowed us to examine performance on related cognitive measures for each profile group. 

## Results

### Cluster Analysis

The four screening variables, Sentence Recall, Math Fluency, Sight Word Efficiency, and Phonemic Decoding Efficiency, were entered into a two-step cluster analysis with noise handling set to the 25% default and using the log-likelihood distance measure. The autoclustering statistics include the Schwarz’s Bayesian Information Criterion (BIC), BIC changes, ratio of BIC changes, and ratio of distance measures. Smaller BICs and BIC changes reflect better models and are used to find an initial estimation for the number of clusters. The initial estimate is refined by taking into account the ratio of distance measure, which reflects the greatest change in distance between the two closest clusters in each hierarchical clustering stage (SPSS, 2001). In the current analysis, the absolute value of the BIC declined to five clusters although the ratio of distance measures indicated that the complexity beyond three clusters is not necessary. Given our interest in identifying the largest number of unique clusters that would fit our data, we repeated the two-step cluster analysis requesting increasing numbers of clusters from three until profiles were duplicated. It should be noted that the order of case entry can influence cluster formation in these analyses. We validated our clustering by repeating the analyses with four additional uniquely randomized case orders and found no additional unique profiles. 

**Figure 1:**
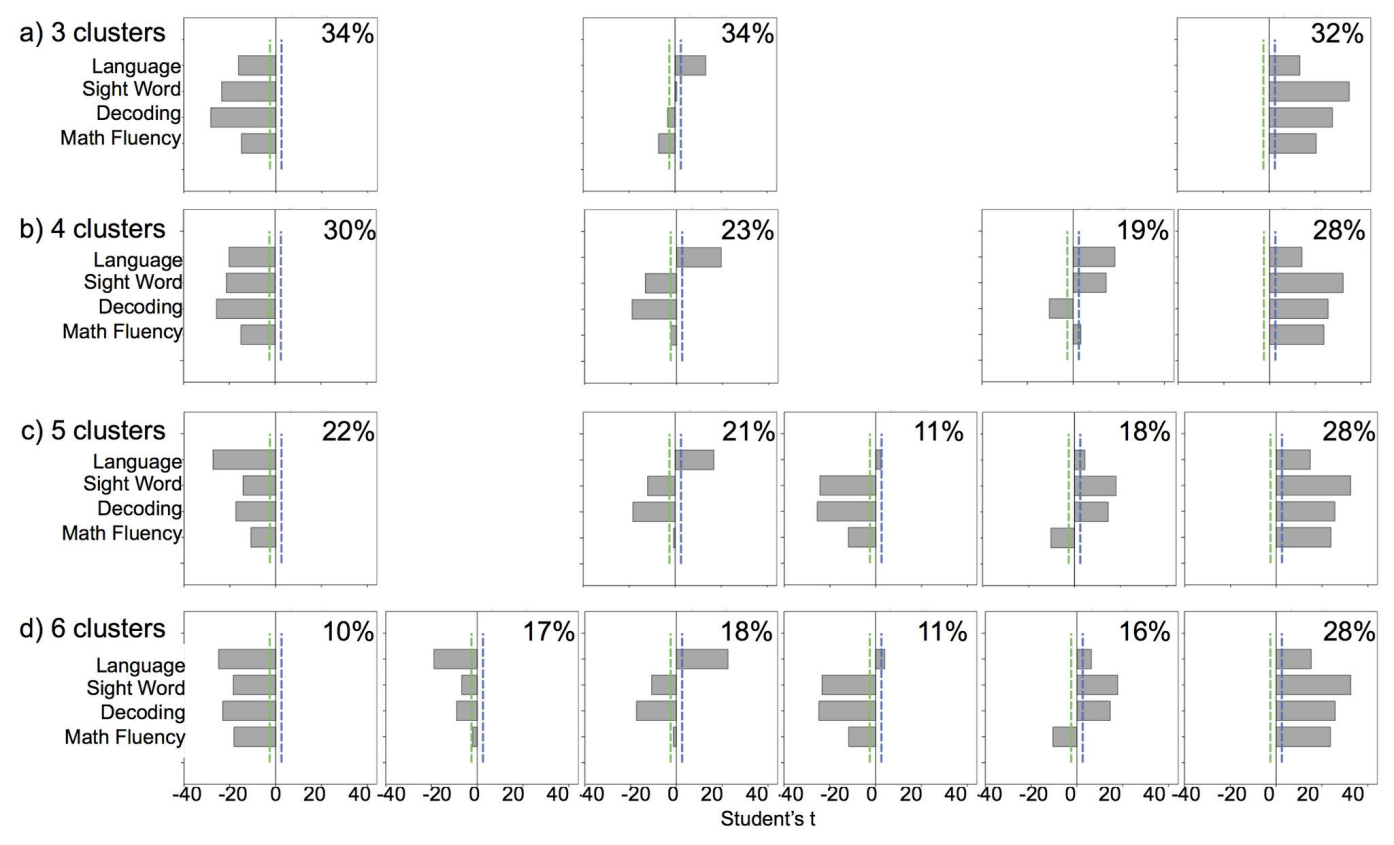
Attribute importance for possible cluster solutions (Language – sentence recall; Sight Word – sight word efficiency; Decoding – phonemic decoding efficiency). Dashed lines represent critical *t*-values at the .05 level.

Results of the cluster analyses are displayed in Figure 1, which presents the percent of participants included in each cluster and the Student’s *t* statistics reflecting each variable’s importance to each cluster. In comparison to the critical value line, the *t*-values provide a guideline as to how each variable contributes to the formation of the cluster and how individual clusters differ from the overall average. As can be seen in Figure 1, the model with 3-clusters selected by the autoclustering procedure included clusters of children distinguished by below average performance overall (34%), above average performance overall (32%), and largely average performance with above average sentence recall and below average math fluency scores (average with language strength; 34%). Analyses were discontinued at 7 clusters due to the presence of two clusters with the same profile: positive and significant *t*-values on all four measures. The most complex solution of unique clusters, then, was the 6-cluster model, which included both overall below (cluster 1) and above average profiles (cluster 6), as well as separable profiles for below average reading efficiency (both sight word and phonemic decoding efficiency; cluster 3), below average math fluency (cluster 5), below average math fluency and reading efficiency (cluster 4), and below average sentence recall and reading efficiency (cluster 2). 

### Descriptive Statistics for Clusters

Table 1 presents descriptive statistics for the 6-cluster solution including cluster size, sex distribution, age, screening measure centroids and standard deviations, and cluster descriptors. The mean ages across clusters varied by five months with cluster 5 (below average math fluency) and cluster 6 (above average overall) tending to be comprised of older children. While significant age differences did occur in this large sample, effect sizes did not exceed 0.02 indicating a very small effect of chronological age on the clustering. There were no significant sex differences in the 6-cluster model, X^2^(5) = 9.7, *p* = .082. In the 3-cluster model selected by the autoclustering solution (see Figure 1), however, more males comprised the below average overall cluster while more females comprised the average with language strength cluster, X^2^(2) = 8.6, *p* = .014. 

**Table 1:** Descriptive statistics for 6 clusters (means and standard deviations).

Cluster	n	No. males	Age (mths)	SR	SWE	PDE	MF	Cluster Descriptor
1	111	63	93.3 (12.4)	73.3 (11.4)	81.0 (10.9)	83.1 (7.8)	83.2 (9.7)	Below average overall
2	188	102	93.7 (13.9)	86.1 (10.2)	96.6 (7.2)	95.0 (7.9)	98.3 (11.3)	Below average sentence recall
3	202	108	94.1 (14.4)	109.6 (6.0)	95.7 (5.9)	93.0 (5.8)	99.0 (10.6)	Below average reading efficiency
4	120	74	95.0 (13.6)	102.8 (7.3)	79.1 (9.9)	82.7 (7.7)	89.6 (9.7)	Below average math and reading
5	186	83	98.3 (12.7)	104.5 (9.6)	108.5 (6.4)	107.4 (6.9)	94.0 (7.8)	Below average math fluency
6	313	179	96.7 (12.3)	108.3 (9.4)	115.1 (8.1)	116.1 (11.0)	115.1 (11.3)	Above average overall

**Figure 2:**
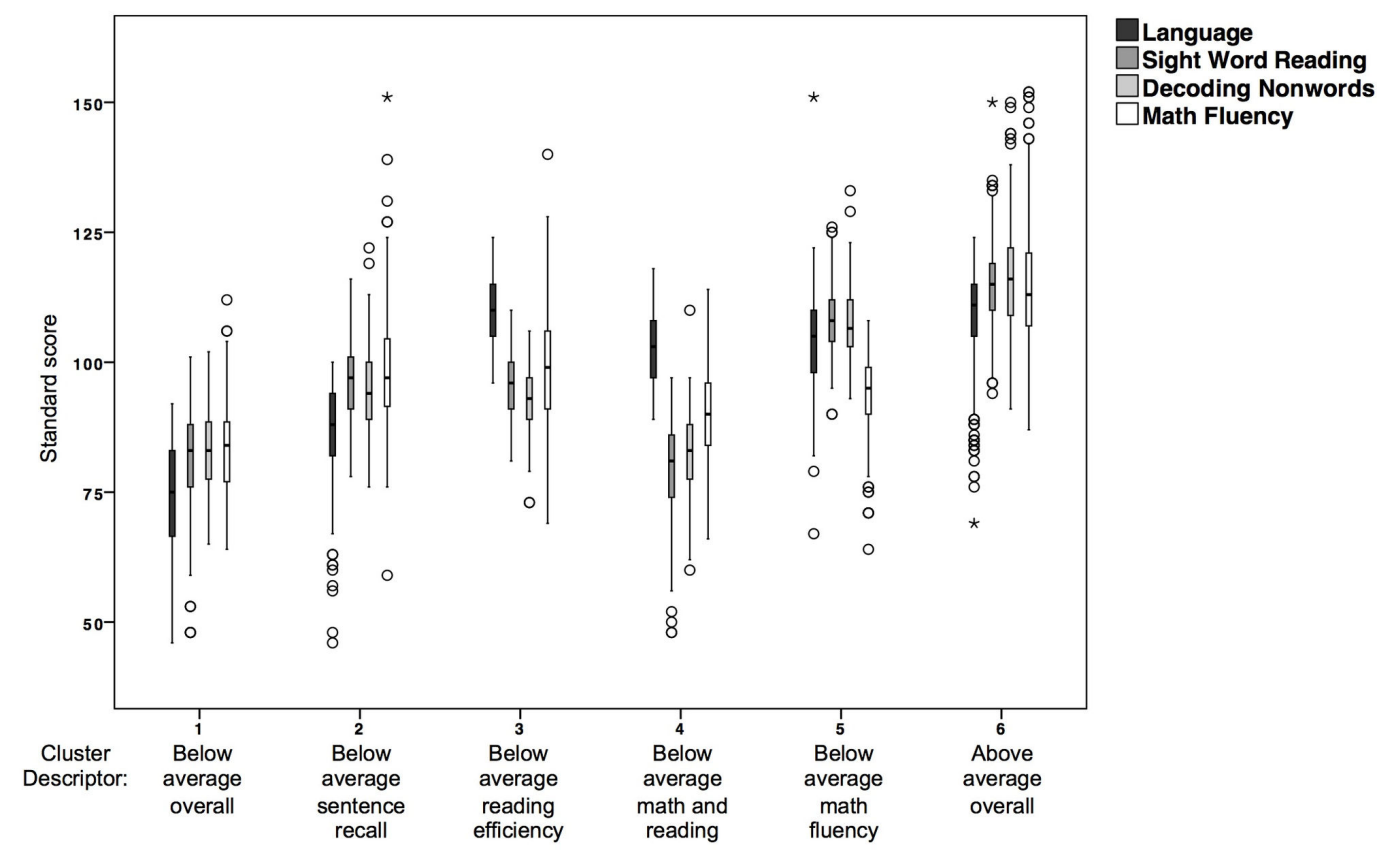
Boxplot for screening measures for each cluster in the 6-cluster solution (Language – sentence recall; Sight Word – sight word efficiency; Decoding – phonemic decoding efficiency). Solid line marks standard score of 100, and dashed line marks standard score of 85.

The boxplot presented in Figure 2 compares performance on all screening measures for the 6-cluster model. The number of outliers was greatest for the sentence recall measure. Clusters 1, 2, and 6 have a pattern of low sentence recall compared to stronger and similar word and nonword reading efficiency and math fluency. These clusters (1,2,6) are distinguished by overall performance (low, average, high, respectively). Clusters 3 and 4 show the opposite pattern with stronger sentence recall performance compared to reading and math performance. Finally, cluster 5 is characterized by lower math than sentence recall or reading scores. 

### Cluster Validation

In order to validate our clusters, the same pattern of performance across clusters would need to be demonstrated for additional measures of language, reading, and math such as those available for our Standardized Test Subsample. Thus, we compared the standardized test performance of our Standardized Test Subsample in a multivariate ANOVA with the Composite Language Score (language), reading fluency, and calculations scores entered as multivariates. The between group factor in this ANOVA was the cluster (6 levels) to which each child had been assigned in our cluster analysis. All effects were significant, *F* (5, 316) > 11.5, *p* < .001, *η* ^2^_p_ > 0.155. Descriptive statistics and results of pairwise comparisons with Bonferroni correction are presented in Table 2. Clusters 1 and 6 (overall below and above average) were readily distinguished in these comparisons by their low vs. high scores, respectively. There was reasonable agreement for two of the other clusters: Cluster 2 (below average sentence recall) was associated with lower language than reading fluency scores and cluster 4 (below average math fluency and reading efficiency) was associated with higher language than reading fluency and calculations scores. Numerically, results corresponded for cluster 5 (below average math fluency) with the math calculations score lower than scores on the language or reading fluency measures for this group, however this difference was not significant. The pattern for the remaining cluster, cluster 3 (below average reading efficiency), did not match the cluster profile in that the reading fluency score was not disproportionately lower. On these additional tests, cluster 3 presented with average test scores on all measures (average overall). 

**Table 2:** Descriptive statistics and results for pairwise comparison in the cluster validation.

Cluster	Cluster Descriptor	n	CLS	Reading Fluency	Calculations	Significant differences across clusters	Validation Descriptor	Matches model?
1	Below average overall	69	83.4 (11.3)	87.1 (10.7)	80.1 (18.2)	CLS < all others; RF & Calc < all but cluster 4	Below average overall	✓
2	Below average sentence recall	63	93.8 (11.7)	102.4 (9.5)	95.5 (19.4)	CLS >1 & < 3, 5, 6; RF > 1, 4 & < 6	Lower language than reading	✓
3	Below average reading efficiency	70	104.0 (10.8)	101.6 (9.5)	95.9 (15.0)	CLS > 1, 2, 4 & < 6; RF > 1, 4 & < 6	Average overall	✗
4	Below average math and reading	38	93.8 (11.9)	88.3 (15.7)	88.0 (18.0)	CLS > 1 & < 3, 5, 6; RF < 2, 3, 5, 6; Calc < 6	Below average reading (and math)	✓
5	Below average math fluency	44	104.8 (10.8)	106.2 (8.3)	96.7 (14.8)	CLS > 1, 2, 4; RF > 1, 4	Above average language & reading (not math)	?
6	Above average overall	38	107.2 (11.0)	109.3 (10.3)	101.4 (10.8)	CLS & RF > 1, 2, 3, 4; Calc > 1, 4	Above average overall	✓

### Associations with Related Measures

Given the reasonable validation of our clusters in the Standardized Test Subsample as described above, we next explored differences in performance on our cognitive measures across clusters. To do this, we completed a multivariate ANOVA with cluster (6 levels) as the between group factor, and the data related to short-term memory, working memory, intelligence, and phonological awareness from our Standardized Test Subsample entered as multivariates. All effects were significant, *F* (5, 300) > 5.9, *p* < .001, *η* ^2^_p_ > 0.09, all cases. Descriptive statistics and results of pairwise comparisons with Bonferroni correction are presented in Table 3. Cluster 1 (below average overall) was consistently distinguished (relative to at least three other clusters) by a significantly below average pattern of performance on all of the measures except block recall (visuospatial short-term memory). Cluster 2 (below average sentence recall) was differentiated by significantly lower phonological short-term memory in the context of average performance on the remaining measures. Cluster 4 (below average reading and math) was associated with lower phonological awareness but average phonological short-term memory. Clusters 5 (above average except math) and 6 (above average overall) were characterized by higher performance IQ. Cluster 3 was not distinguished by any of the test scores. Cluster 3 was identified as ‘below average reading efficiency’ in the cluster analysis, but had average overall performance in the cluster validation. This latter pattern is consistent with the finding that this cluster was not distinguished by any of the related measures. Nevertheless, this group did show high variability on a task closely related to reading, the phonological awareness task with a standard deviation of 6.5, more than 2.5 times that of any of the remaining groups (range of *SD* for remaining groups: 2.4 to 2.6).

**Table 3:** Descriptive statistics and pairwise comparisons for related measures between cluster groupings.

	Cluster / Validation Descriptor	Ph. STM	vssp STM	Working Memory	Peformance IQ	Ph. awareness	Summary of consistent differences relative to 3 or more other clusters
1	Below average overall / Below average overall	85.5^bcde^ (11.9)	96.4^ab^ (17.7)	88.1^abcd^ (10.9)	91.4^abcd^ (9.7)	8.0^abcd^ (2.6)	phonological STM, working memory, performance IQ, phonological awareness significantly lower
2	Below average sentence recall / Lower language than reading	88.1^fghi^ (9.8)	101.5^c^ (15.3)	94.8^fa^ (9.4)	99.6^aef^ (11.8)	10.8^a^ (2.4)	phonological STM significantly lower
3	Below average reading efficiency / Average overall	98.7^bf^ (12.4)	103.4 (15.0)	97.7^b^ (9.3)	101.4^bg^ (12.8)	11.6^be^ (6.5)	
4	Below average math and reading / Below average reading (and math)	96.7^cg^ (13.5)	99.5^d^ (18.3)	95.4^c^ (8.9)	98.3^hi^ (12.7)	9.1^efg^ (2.5)	phonological awareness significantly lower (with average phonological STM)
5	Below average math fluency / Above average language & reading	101.6^dh^ (14.5)	111.8^acd^ (15.3)	101.4^df^ (10.1)	108.6^ceh^ (16.8)	11.8^cf^ (2.6)	PIQ, visuospatial (vssp) short term memory significantly higher
6	Above average overall / Above average overall	103.4^ei^ (11.8)	107.3^b^ (13.5)	100.6^e^ (9.0)	109.9^dfg^ (13.4)	12.1^dg^ (2.3)	PIQ significantly higher

Note: Same superscript indicates significantly different pairs; verbal STM – digit recall; vssp STM – block recall; Phonological (Ph.) awareness – elision (scaled score: *M* = 10, *SD* = 3).

### Profiles of Children with Impaired Performance on Screening Measures

The distribution of cluster membership across the epidemiological sample is shown in Table 4. Approximately equal proportions (16-18%) of children were included in clusters 2 (below average sentence recall), 3 (below average reading efficiency), and 5 (below average math fluency) with lower proportions (10-11%) included in clusters 1 (below average overall) and 4 (below average reading and math), and a higher proportion in cluster 6 (above average overall: 28%). Thus, 38% of the sample had a general learning profile with largely equivalent performance across measures (clusters 1 or 6), and 62%, a more specific learning profile with marked differences in performance on at least one measure (clusters 2, 3, 4, and 5). 

**Table 4:** Distribution of cluster membership (percentage) for study subsamples.

	Cluster Descriptor / Validation Descriptor^a^	Epidemiological Sample	Children with potential LD^b^
1	Below av. Overall	10%	27%
2	Below av. sentence recall / Lower language than reading	17%	24%
3	Below av. reading efficiency / Av. Overall	18%	12%
4	Below av. math and reading / Below av. reading (and math)	11%	27%
5	Below av. math fluency / Above av. language and reading	16%	7%
6	Above av. Overall	28%	3%

*Note*: a – Validation descriptor shown if different from cluster descriptor; b – children who scored < 1 *SD* below standardized mean on at least one screening measure; av. – average

A final descriptive analysis considered whether the learning profile distributions of children with a potential learning disability differed from that of the Epidemiological Sample. To do this, we compared the cluster membership distribution (see Table 4) in the Epidemiological Sample to that of children for whom we had some evidence of a learning disability as reflected by performance of at least 1 *SD* below the mean on at least one screening measure (Sentence Recall, Sight Word Reading Efficiency, Phonemic Decoding Efficiency, Math Fluency). The cutoff criterion of -1 *SD*, conventionally considered to reflect a large effect size (Cohen, 1988), was chosen to capture all children who performed poorly on at least one screening measure. Note that this cutoff criterion differs from that described for the Standardizd Test Subsample, which was motivated by other studies and not suited to the research question being addressed in this analysis. The proportions of children with a general learning profile was smaller for the potential learning disability group (clusters 1 and 6: 30 vs. 38%, for the potential learning disability and Epidemiological Samples, respectively), and included a higher proportion of below average profiles (cluster 1: 27% vs. 10%). A higher proportion of the potential learning disability group had a specific learning profile (clusters 2, 4, and 5: 70% vs. 58%). Comparatively high proportions characterized the potential learning disability group for clusters 2 (below average sentence recall: 24 vs. 17%) and 4 (below average reading and math 4: 27 vs. 11%) whereas smaller proportions were found for clusters 3 (below average reading efficiency: 12% vs. 18%) and 5 (below average math fluency: 7% vs. 16%).

## Discussion

In a large epidemiological sample, we identified clusters of children differing in patterns of relative strengths and weaknesses in language, reading, and math. In addition to finding profiles of children who had globally above or below average abilities across all three academic skills, we found separable profiles of children who had relative weaknesses specific only to language, reading efficiency, math fluency, or reading and math combined. Using independent measures available for a subsample of the original participant group, we validated all profiles except that involving relative weaknesses in reading efficiency. Examination of the learning profiles of the subset of the epidemiological group for whom there was some evidence of a learning disability (i.e., below average performance on one or more screening measures) revealed higher proportions of relatively specific deficits in language, or reading and math. Importantly, we discovered that these unique learning profiles could be further distinguished by differing abilities in underlying cognitive processes including immediate memory, intelligence, and phonological awareness. Perhaps not surprisingly, the overall below average group was weak across all of these cognitive processes, and higher nonverbal intelligence scores characterized the above average overall group. Of interest, limitations in phonological short-term memory characterized the group with a relative weakness in language, whereas limitations in phonological awareness were observed in the group with dual reading and math weaknesses. 

The present findings clearly establish that there are patterns in children’s learning that go beyond a simple below average, average, above average grouping. Using a novel cluster analysis approach, we identified children with both general and specific learning profiles. Over one third of our large epidemiological sample had a general learning profile characterized by either consistently above or below average performance across all measures. These groups with generally enhanced or depressed learning were further distinguished by significantly higher or lower nonverbal intelligence, respectively, a finding consistent with the well-established relationship between general intellectual ability and academic performance (e.g., [77,78]). 

Of greater interest are the four specific learning profiles we observed characterized by below average scores on one of language, reading and math, reading only, or math only. Our validation analysis using independent measures in a subset of the original sample confirmed the first two profiles, and was numerically consistent for the group with a specific math learning difficulty. There was less evidence for a specific reading difficulty in that this profile was not confirmed in our validation analysis using an additional reading fluency measure that involved reading short sentences. Nevertheless, it may be that the reading fluency task was not as sensitive to individual differences in reading as the single word and nonword reading measures employed in our cluster analysis. The results of this validation analysis must be interpreted with caution because the subsample on which it was based differed in composition from the original epidemiological sample. Nevertheless, the considerable consistency in the learning characteristics between the two independent sets of measures suggests that the learning profiles identified in our cluster analysis warrant further attention.

Our analysis of children who performed poorly on the screening measures provided unique information about the learning profiles of children with possible learning disabilities. Just over one quarter (27%) of these children exhibited a general pattern of poor scores across measures, compared to 10% in the entire sample. Importantly, 70% presented with a relatively specific learning impairment. These data are the first to suggest that specific patterns in learning strengths and weaknesses characterize the majority of children with learning disabilities. 

What does the observed comorbidity tell us about potential underlying factors? Consider first the pattern observed in the present results for the oral language measure, sentence recall. Poor language coupled with somewhat higher and similar scores on reading and math occurred across three clusters and characterized 45% of the entire sample. Low language scores never occurred entirely in isolation. They occurred either with below average reading only (relative to the sample), or, in more severe cases, both below average reading and math (i.e., a general below average profile). Thus, poor language was associated with below average reading consistently, but was linked to low math scores only when language scores were markedly poor. These language-based clusters were differentiated in our analysis of related cognitive measures by low phonological short-term memory, a finding consistent with previous research demonstrating strong links between phonological short-term memory and vocabulary development [79,80], and poor phonological short-term memory and SLI [81,82]. Taken together, this pattern of results suggests that a primary language impairment may underlie deficits in the other academic domains for these language-based clusters. 

Reduced efficiencies in reading occurred either with a language deficit, with a math deficit, or with no other deficits. The two comorbid clusters (i.e., weaknesses in reading and language, or in reading and math) were differentiated by their cognitive profile: the below average reading and language cluster was associated with poor phonological short-term memory, whereas the below average math and reading cluster had low phonological awareness. These differing cognitive profiles may suggest different underlying causes, the language impairment in the case of the below average reading and language cluster with low phonological short-term memory, and a deficit specific to another aspect of phonological processing for the below average reading and math cluster. Although less clear, the results for the reduced reading efficiency cluster revealed high variability in phonological awareness potentially indicating some phonological processing weakness in this group as well. The common cognitive profile of low/variable phonological awareness in the low reading only and low reading and math clusters suggests a possible common etiology to this reading impairment that is distinct from the mechanism involved in the below average language and reading cluster. Certainly, the finding of a specific association between phonological awareness and reading is consistent with many previous studies of typical reading development [83] and dyslexia [18].

For math fluency, below average scores occurred in relative isolation, with below average reading efficiency, or with a general below average profile (including markedly low language). The comorbid clusters with math were differentiated by their cognitive profiles with the below average reading and math cluster having a phonological awareness deficit, and the general below average group having multiple deficits. Importantly, the cognitive profile of the general below average cluster had an impairment in common with the other clusters involving below average language, phonological short-term memory. Once again, these results suggest distinct etiologies for these two comorbid deficits, one possibly language-based in the general below average profile, and one related to reading but not language. Unfortunately, our measures did not capture any cognitive deficits in our below average math fluency cluster. The only indication of a difference between our below average math only vs. below average math and reading clusters was that the below average math and reading cluster had a phonological awareness deficit while the below average math fluency group did not. It may be that differences in these groups would have been revealed had additional cognitive measures been included. In future, studies of this nature should include measures specific to the cognitive mechanisms thought to support math skills such as estimating the number of objects in a group [23], or comparing quantities [24,25].

While it is interesting to speculate on the patterns observed in the present study, it is clear that caution is warranted in interpreting the observed comorbidity. For one, the 3-cluster solution adequately explained the data. It may be that a general factor can capture a considerable proportion of the variation characterizing young children’s learning. Indeed, our reading and math measures were timed placing demands on processing speed, which has been suggested as a common deficit in reading disorder and ADHD [84]. Nevertheless, we ensured that our clustering was reflective of the data by completing several runs with different ordering of the data. We considered it important to examine all of the unique learning profiles present in the data, and so explored additional clusters until no further unique profiles were identified. Importantly, the character of these results did not change when we used these different approaches, supporting the view that the clusters were robust. As well, the epidemiological sample in the present study was relatively small for a population-based sample. Replication of the cluster analysis with a larger sample is needed to better establish the learning profiles. Finally, we used single measures to estimate language, reading, and math skills, which could have influenced our results. Although we employed valid and reliable measures commonly used in identifying language, reading, and math disabilities, the use of a single indictor per construct is not ideal. It is possible too that the particular indicator influenced the cluster characteristics. For example, there are phonological short-term memory demands associated with sentence recall, the task that indexed language skills in the present study. It may be no surprise, then, that the weak language clusters were associated with low phonological short-term memory. It is clear that future studies should include multiple measures of each of these complex skills in order to provide a more robust estimation of these abilities. Further research should also include additional cognitive measures administered to all individuals in the epidemiological sample in order to better understand comorbid learning disabilities in children. 

## Conclusion

The present study examined learning profiles on language, reading, and math screening measures across a large epidemiological sample of school age children. Three primary clusters reflective of below average, largely average, and above average performance across measures were sufficient to describe the sample. More detailed analyses identified overall above and below average profiles, as well as unique learning profiles involving weaknesses in language, reading, math, or reading and math. These latter four specific profiles characterized 70% of those with a potential learning disability as evidenced by below average performance on at least one screening measure. As well, differences in cognitive profiles characterized several of the clusters including associations between poor phonological short-term memory and language-based weaknesses, and between poor phonological awareness and reading weaknesses. The results have implications for the study of learning disabilities that warrant further investigation and replication. Specifically, distinct specific and cormorbid subtypes of learning profiles were identified and were common among those with potential learning disabilities. As well, the findings suggest different etiologies for language-based deficits across domains, reading-related impairments in reading and math, and isolated math disabilities.

## References

[1] Leonard L (2004) Specific language impairment in children. In: Kent RD The MIT encyclopedia of communication disorders. Cambridge, MA: MIT Press pp. 402-405.

[2] van Weerdenburg M, Verhoeven L, Balkom HV (2006) Towards a typology of specific language impairment. J Child Psychol Psychiatry 47: 176-189. doi:10.1111/j.1469-7610.2005.01454.x. PubMed: 16423149. 16423149

[3] Leinonen S, Muller K, Lappanen PHT, Aro M, Ahonen T et al. (2001) Heterogeneity in adult dyslexic readers: Relating processing skills to the speed and the accuracy of oral text reading. Read Writ 14: 265-296. doi:10.1023/A:1011117620895.

[4] Murphy L, Pollatsek A (1994) Developmental dyslexia: Heterogeneity without discrete subgroups. Ann Dyslexia 44: 120-146. doi:10.1007/BF02648158. 24234049

[5] Rubinsten O, Henik A (2009) Developmental dyscalculia: Heterogeneity might not mean different mechanisms. Trends Cogn Sci 13: 92-99. doi:10.1016/j.tics.2008.11.002. PubMed: 19138550. 19138550

[6] Bishop DVM, Rutter M (2008) Neurodevelopmental disorders: Conceptual issues. In: Rutter M Bishop D Pine D Scott S Stevenson J Rutter’s child and adolescent psychiatry, 5th ed. Oxford, UK: Blackwell Publishing House pp. 32-42.

[7] Pennington BF, Willcutt E, Rhee SH (2005) Analyzing comorbidity. In: Kail R Advances in Child Development and Behavior. vol. 33 San Diego, CA: Elsevier pp. 264-304. 10.1016/s0065-2407(05)80010-2 16101120

[8] Trauner D, Wulfeck B, Tallal P, Hesselink J (2000) Neurologic and MRI profiles of language impaired children. Dev Med Child Neurol 46: 470-475. doi:10.1017/S0012162200000876. 10972419

[9] Lahey M, Edwards J (1996) Naming errors of children with specific language impairment. J Speech Lang Hear Res 42: 195-205. PubMed: 10025554. 10.1044/jslhr.4201.195 10025554

[10] Rice RL, Wexler K, Cleave PL (1995) Specific language impairment as a period of extended optional infinitive. J Speech Lang Hear Res 38: 850-863. PubMed: 7474978. 10.1044/jshr.3804.850 7474978

[11] van der Lely HKJ (1996) Specifically language impaired and normally developing children: Verbal passive vs. adjectival passice sentence interpretation. Lingua 98: 243-272 doi:10.1016/0024-3841(95)00044-5.

[12] Rice ML (2003) A unified model of specific and general language delay: Grammatical tense as a clinical marker of unexpected variation. In Levy Y Schaeffer J Language competence across populations: Toward a definition of specific language impairment. Mahwah, NJ: Erlbaum pp. 63–94.

[13] Bishop DVM, North T, Donlan C (1996) Nonword repetition as a behavioural marker for inherited language impairment: Evidence from a twin study. J Child Psychol Psychiatry 37: 391-403. doi:10.1111/j.1469-7610.1996.tb01420.x. PubMed: 8735439. 8735439

[14] Conti-Ramsden G (2003) Processing and linguistic markers in young children with specific language impairment (SLI). J Speech Lang Hear Res 46: 1029-1037. doi:10.1044/1092-4388(2003/082). PubMed: 14575341. 14575341

[15] Frith U (1985) Beneath the surface of developmental dyslexia. In: Patterson K Marshall J Coltheart M Surface dyslexia, neuropsychological and cognitive studies of phonological reading. London: Erlbaum pp 301-330.

[16] Pollatsek A, Lesch M, Morris RK, Rayner K (1992) Phonological codes are used in integrating information across saccades in word identification and reading. J Exp Psychol Hum Percept Perform 18: 148-162. doi:10.1037/0096-1523.18.1.148. PubMed: 1532185. 1532185

[17] Wagner RK, Torgesen JK (1987) The nature of phonological processing and its causal role in the acquisition of reading skills. Psychol Bull 101: 192-212. doi:10.1037/0033-2909.101.2.192.

[18] Torgensen JK, Al Otaiaba S, Grek M (2004) Assessment and instruction for phonemic awareness and word reading skills. In: Catts HW Kamhi AG Language basis of reading disabilities, 2nd ed. Needham Heights, MA: Allyn & Bacon.

[19] Butterworth B (2002) Screening for dyscalculia: A new approach. Paper Presented at the Mathematical Difficulties: Psychology, Neuroscience, and Interventions Conference. UK: Oxford . Available: www.mathematicalbrain.com/pdf/.

[20] Geary DC (1993) Mathematical disabilities: cognitive, neuropsychological, and genetic components. Psychol Bull 114: 345-362. doi:10.1037/0033-2909.114.2.345. PubMed: 8416036. 8416036

[21] Dehaene S (1997) The Number Sense: How the Mind Creates Mathematics. NY, New York: Oxford University Press.

[22] Dehaene S (2001) Précis of the number sense. Mind Lang 16: 16-36. doi:10.1111/1468-0017.00154.

[23] Schleifer P, Landerl K (2011) Subitizing and counting in typical and atypical development. Dev Sci 14: 280-291. doi:10.1111/j.1467-7687.2010.00976.x. PubMed: 22213901. 22213901

[24] Landerl K, Bevan A, Butterworth B (2004) Developmental dyscalculia and basic numerical capacities: a study of 8-9-year-old students. Cognition 93: 99-125. doi:10.1016/j.cognition.2003.11.004. PubMed: 15147931. 15147931

[25] Mussolin C, Mejias S, Noël MP (2010) Symbolic and nonsymbolic number comparison in children with and without dyscalculia. Cognition, 115: 10-25. doi:10.1016/j.cognition.2009.10.006. PubMed: 20149355. 20149355

[26] Broomfield J, Dodd B (2004) Children with speech and language disability: Caseload characteristics. Int J Lang Commun Disord 39: 303-324. doi:10.1080/13682820310001625589. PubMed: 15204443. 15204443

[27] Tirosh E, Cohen A (1998) Language deficit with attention-deficit disorder: A prevalent comorbidity. J Child Neurol 13: 493-497. doi:10.1177/088307389801301005. PubMed: 9796755. 9796755

[28] Hill EL (2001) Non-specific nature of specific language impairment: A review of the literature with regard to concomitant motor impairments. Int J Lang Commun Disord 36: 149-171. doi:10.1080/13682820010019874. PubMed: 11344592. 11344592

[29] Ramus F (2003) Developmental dyslexia: Specific phonological deficit or general sensorimotor dysfunction? Curr Opin Neurobiol 13: 212-218. doi:10.1016/S0959-4388(03)00035-7. PubMed: 12744976. 12744976

[30] Germanò E, Gagliano A, Curatolo P (2010) Comorbidity of ADHD and dyslexia. Dev Neuropsychol, 35: 475-493. doi:10.1080/875656412010494748. PubMed: 20721770. 20721770

[31] Everatt J, Bradshaw MF, Hibbard PB (1999) Visual processing and dyslexia. Perception, 28: 243-254. doi:10.1068/p2743. PubMed: 10615463. 10615463

[32] Mayes SD, Calhoun SL (2006) WISC-IV and WISC-III profiles in children with ADHD. J Atten Disord 9: 486-493. doi:10.1177/1087054705283616. PubMed: 16481665. 16481665

[33] Gross-Tsur V, Manor O, Shalev RS (1996) Developmental dyscalculia: Prevalence and demographic features. Dev Med Child Neurol 38: 25-33. doi:10.1111/j.1469-8749.1996.tb15029.x. PubMed: 8606013. 8606013

[34] McLean AF, Hitch GJ (1999) Working memory impairments in children with specific arithmetic learning difficulties. J Exp Child Psychol 74: 240-260. doi:10.1006/jecp.1999.2516. PubMed: 10527556. 10527556

[35] Hill EL (2001) Non-specific nature of specific language impairment: A review of the literature with regard to concomitant motor impairments. Int J Lang Commun Disord 36: 149-171. doi:10.1080/13682820010019874. PubMed: 11344592. 11344592

[36] Pennington BF (2006) From single to multiple deficit models of developmental disorders. Cognitio, 101: 385-413. doi:10.1016/j.cognition.2006.04.008. PubMed: 16844106. 16844106

[37] McArthur GM, Hogben JH, Edwards VT, Heath SM, Mengler ED (2000) On the “specifics” of specific reading disability and specific language impairment. J Child Psychol Psychiatry 41: 869-874. doi:10.1111/1469-7610.00674. PubMed: 11079429. 11079429

[38] Bishop DVM (2001) Genetic influences on language impairment and literacy problems in children: Same or Different? J Child Psychol Psychiatry 42: 189-198. doi:10.1111/1469-7610.00710. PubMed: 11280415. 11280415

[39] Flax JF, Realpe-Bonilla T, Hirsch LS, Brzustowicz LM, Bartlett CW et al. (2003) Specific language impairment in families: Evidence for co-occurrence with reading impairments. J Speech Lang Hear Res 46: 530-543. doi:10.1044/1092-4388(2003/043). PubMed: 14696984. 14696984

[40] DeThorne LS, Hart SA, Petrill SA, Deater-Deckard K, Thompson LA et al. (2006) Children’s history of speech-language difficulties: Genetic influences and associations with reading-related measures. J Speech Lang Hear Res 49: 1280-1293. doi:10.1044/1092-4388(2006/092). PubMed: 17197496. 17197496 PMC2659564

[41] Newbury DF, Paracchini S, Scerri TS, Winchester L, Addis L et al. (2011) Investigation of dyslexia and SLI risk variants in reading- and language-impaired subjects (2011). Behav Genet 41: 90-104. doi:10.1007/s10519-010-9424-3. PubMed: 21165691. 21165691 PMC3029677

[42] Rice ML, Smith SD, Gayán J (2009) Convergent genetic linkage and associations to language, speech, and reading measures in families of probands with specific language impairment. J Neurodev Disord, 1: 264-282. doi:10.1007/s11689-009-9031-x. PubMed: 19997522. 19997522 PMC2788915

[43] Robertson EK, Joanisse MF (2010) Spoken sentence comprehension in children with dyslexia and language impairment: The roles of syntax and working memory. Appl Psycholinguist 31: 141-165. doi:10.1017/S0142716409990208.

[44] Robertson EK, Joanisse MF, Desroches AS, Ng S (2009) Categorical speech perception deficits distinguish language and reading impairments in children. Dev Sci 12: 753-767. doi:10.1111/j.1467-7687.2009.00806.x. PubMed: 19702768. 19702768

[45] Bishop DVZ, Snowling MJ (2004) Developmental dyslexia and specific language impairment: Same or different? Psychol Bull 130: 858-886. doi:10.1037/0033-2909.130.6.858. PubMed: 15535741. 15535741

[46] Snowling MJ (2000) From language to reading and dyslexia. Dyslexia, 7: 37-46. doi:10.1002/dys.185. 11305230

[47] Bishop DVM, Adams C (1990) A prospective study of the relationship between specific language impairment, phonological disorders, and reading retardation. J Child Psychol Psychiatry 31: 1027-1050. doi:10.1111/j.1469-7610.1990.tb00844.x. PubMed: 2289942. 2289942

[48] Lewis C, Hitch GJ, Peter W (1994) The prevalence of specific arithmetic difficulties and specific reading difficulties in 9- to 10-year-old by and girls. J Child Psychol Psychiatry 35: 283-292. doi:10.1111/j.1469-7610.1994.tb01162.x. PubMed: 8188799. 8188799

[49] Badian NA (1999) Persistent arithmetic, reading, or arithmetic and reading disability. Ann Dyslexia 49: 43-70. doi:10.1007/s11881-999-0019-8.

[50] Jordan NC, Hanich LB (2000) Mathematical thinking in second-grade children with different forms of LD. J Learn Disabil 6: 567-578. doi:10.1177/002221940003300605. PubMed: 15495398. 15495398

[51] Jordan NC, Hanich LB, Kaplan D (2003) A longitudinal study of mathematical competencies in children with specific mathematics difficulties versus children with comorbid mathematics and reading difficulties. Child Dev 7: 834-850. doi:10.1111/1467-8624.00571. PMC2791887 12795393

[52] Jordan NC, Montani TO (1997) Cognitive arithmetic and problem solving: A comparison of children with specific and general mathematics difficulties. J Learn Disabil 30: 624-634. doi:10.1177/002221949703000606. PubMed: 9364900. 9364900

[53] Rubinsten O, Henik A (2006) Double dissociation of functions in developmental dyslexia and dyscalculia. J Educ Psychol 98: 854-867. doi:10.1037/0022-0663.98.4.854.

[54] Fuchs LS, Fuchs D (2002) Mathematical problem-solving profiles of students with mathematics disabilities with and without comorbid reading disabilities. J Learn Disabil 35: 564-574. doi:10.1177/00222194020350060701. PubMed: 15493253. 15493253

[55] Willburger E, Fussenegger B, Moll K, Wood G, Landerl K (2008) Naming speed in dyslexia and dyscalculia. Learn Individ Differ 18: 224-236. doi:10.1016/j.lindif.2008.01.003.

[56] Landerl K, Fussenegger B, Moll K, Willburger E (2009) Dyslexia and dyscalculia: Two learning disorders with different cognitive profiles. J Exp Child Psychol 103: 309-342. doi:10.1016/j.jecp.2009.03.006. PubMed: 19398112. 19398112

[57] Rourke BP (1993) Arithmetic disabilities, specific and otherwise: A neuropsychological perspective. J Learn Disabil 26: 214-216. doi:10.1177/002221949302600402. PubMed: 8515186. 8515186

[58] Rourke BP, Conway JA (1997) Disabilities of arithmetic and mathematical reasoning: Perspectives from neurology and neuropsychology. J Learn Disabil 30: 34-36. doi:10.1177/002221949703000103. PubMed: 9009877. 9009877

[59] Rourke BP, Finlayson AJ (1978) Neuropsycholgical significant of variations in patterns of academic performance: Verbal and visual-spatial abilities. J Abnorm Child Psychol 6: 121-133. doi:10.1007/BF00915788. PubMed: 632453. 632453

[60] Geary DC (2004) Mathematics and Learning Disabilities. J Learn Disabil 37: 4-15. doi:10.1177/00222194040370010201. PubMed: 15493463. 15493463

[61] Shalev RS, Manor O, Gross-Tsur V (1997) Neuropsychological aspects of developmental dyscalculia. Math Cogn, 3: 105-120. doi:10.1080/135467997387434.

[62] Dehaene S, Piazza M, Pinel P, Cohen L (2003) Three parietal circuits for number processing. Cogn Neuropsychol 20: 487-506. doi:10.1080/02643290244000239. PubMed: 20957581. 20957581

[63] Arvedson PJ (2002) Young children with specific language impairment and their numerical cognition. J Speech Lang Hear Res 45: 970-982. doi:10.1044/1092-4388(2002/079). PubMed: 12381054. 12381054

[64] Donlan C, Bishop DVM, Hitch GJ (1998) Magnitude comparisons by children with specific language impairments: Evidence of unimpaired symbolic processing. Int J Lang Commun Disord 33: 149-160. doi:10.1080/136828298247802. PubMed: 9709434. 9709434

[65] Fazio BB (1994) The counting abilities of children with specific language impairment: A comparison of oral and gestural tasks. J Speech Hear Res 37: 358-368. PubMed: 8028317. 8028317 10.1044/jshr.3702.358

[66] Fazio BB (1996) Mathematical abilities of children with specific language impairment: A 2-year follow-upJ. J Speech Lang Hear Res 39: 839-849. 10.1044/jshr.3904.839 8844563

[67] Kleemans T, Segers E, Verhoeven L (2011) Cognitive and linguistic precursors to numeracy in kindergarten: Evidence from first and second language learners. Learn Individ Differ 21: 555-561. doi:10.1016/j.lindif.2011.07.008.

[68] Redmond SM (2005) Differentiating SLI from ADHD using children's sentence recall and production of past tense morphology. Clin Linguist Phon 19: 109-127. doi:10.1080/02699200410001669870. PubMed: 15704501. 15704501

[69] Archibald LMD, Joanisse MF (2009) On the sensitivity and specificity of nonword repetition and sentence recall to language and memory impairments in children. J Speech Lang Hear Res 52: 899-914. doi:10.1044/1092-4388. PubMed: 19403945 (2009/08-0099) 19403945

[70] Woodcock RW, McGrew KS, Mather N (2001) Woodcock-Johnson Tests of Achievement. Itasca, IL: Riverside Publishing.

[71] Torgensen JK, Wagner RK, Rashotte CA (1999) Test of Word Reading Efficiency. AGS Publishing.

[72] Records NL, Tomblin JB (1994) Clinical decision making: Describing the decision rules of practicing speech-language pathologists. J Speech Lang Hear Res 37: 144-156. PubMed: 8170120. 8170120

[73] Semel E, Wiig EH, Secord WA (2003) Clinical Evaluation of Language Fundamentals, fourth Edition. Toronto, Canada: The Psychological (p. CELF-4) Corporation/A Harcourt: Assessment Company.

[74] Wagner RK, Torgensen JK, Rashotte CA (1999) Comprehensive test of phonological processes. Austin, Texas: Pro-Ed.

[75] Wechsler D (1999) Wechsler Abbreviated Scale of Intelligence (WASI). San Antonio, TX: Psychological Corporation.

[76] Alloway TP (2007) The Automated Working Memory Assessment. London: Harcourt Publishing House Assessment

[77] Funham A, Monsen J (2009) Personality traits and intelligence predict academic school grades. Learn Individ Differ 19: 28-33. doi:10.1016/j.lindif.2008.02.001.

[78] Chamorro-Premuzic T, Furnham A (2005) Personality and intellectual competence. Mahwah, NJ: Lawerence Erlbaum Associates Inc.

[79] Gathercole SE, Service E, Hitch GJ, Adams A, Martin AJ (1999) Phonological short-term memory and vocabulary development: Further evidence on the nature of the relationship. Appl Cogn Psychol 13: 65-77. doi:10.1002/(SICI)1099-0720(199902)13:1<65::AID-ACP548>3.0.CO;2-O.

[80] Gupta P, MacWhinney B (1997) Vocabulary acquisition and verbal short-term memory: Computational and neural bases. Brain Lang 59: 267-333. doi:10.1006/brln.1997.1819. PubMed: 9299067. 9299067

[81] Archibald LMD, Gathercole SE (2006) Short-term and working memory in specific language impairment. Int J Lang Commun Disord 41: 675-693. doi:10.1080/13682820500442602. PubMed: 17079222. 17079222

[82] Montgomery JW (2004) Sentence comprehension in children with specific language impairment: Effects of input rate and phonological working memory. Int J Lang Commun Disord 39: 115-133. doi:10.1080/13682820310001616985. PubMed: 14660189. 14660189

[83] Lonigan CJ, Burgess SR, Anthony JA (2000) Development of emergent literacy and reading skills in preschool children: Evidence from a latent variable longitudinal study. Dev Psychol 36: 596-613. doi:10.1037/0012-1649.36.5.596. PubMed: 10976600. 10976600

[84] Willcutt EG, Betjemann RS, McGrath LM, Chhabildas NA, Olson RK et al. (2010) Etiology and neuropsychology of comorbidity between RD and ADHD: the case for multiple-deficit models. Cortex 46: 1345-1361. doi:10.1016/j.cortex.2010.06.009. PubMed: 20828676. 20828676 PMC2993430

